# Cataract Surgery in Complex Anterior Segment Pathology

**DOI:** 10.1155/2022/9808735

**Published:** 2022-01-24

**Authors:** Miltos Balidis, Miguel Rechichi, Rajesh Fogla, Zisis Gatzioufas

**Affiliations:** ^1^Ophthalmica Institute, Thessaloniki, Greece; ^2^Centro Polispecialistico Mediterraneo, Selia Marina, Italy; ^3^Cornea Service, Apollo Hospital, Hyderabad, India; ^4^Department of Ophthalmology, University of Basel, Basel, Switzerland

Modern cataract surgery provides a high level of efficacy and safety, incorporating numerous technological innovations, such as femtosecond laser technology or 3D visualization, as well as advances in intraocular lens (IOL) technology with the development of special or premium IOLs, such as aniridia IOLs, toric IOLs, or multifocal IOLs. As a result, unparalleled visual outcomes can be achieved for patients by using customized solutions for each individual patient.

However, there are an increasing number of anterior segment pathologies, which could complicate cataract surgery and compromise the expected visual outcomes. Cataract surgery in the presence of these complex conditions requires a special approach, including modification of surgical techniques and/or the use of special IOLs.

In this special issue, Schmidt et al. provide a systematic review on the results of comparative studies of modern cataract surgery in pediatric uveitis with or without intraocular lens (IOL) implantation and perform comparative meta-analyses to compare visual acuity outcomes and complication rates. Takai et al. compare the refractive status between eyes implanted with toric and nontoric IOLs during combined cataract surgery and microhook ab interno trabeculotomy, a minimally invasive glaucoma surgery. Tsakiris et al. elaborate on surgical and perioperative considerations for the treatment of cataract in eyes with glaucoma. Ahmadzadeh et al. systematically review the literature to compare the efficacy and safety of phacotrabeculectomy and trabeculectomy either alone or followed by later phacoemulsification.

Moreover, Hu et al. describe a flapless/grooveless technique for four-point refixation of a dislocated IOL with four fenestrated haptics, as well as a minimally invasive suture fixation technique for four-point fixation of IOLs in the treatment of aphakic eyes, namely, the intrascleral suture anchoring technique. Wu et al. evaluate the efficacy of a modified four-point fixation technique for the repositioning of a dislocated IOL with four eyelets in the absence of capsule support. Finally, Iyer et al. highlight the nuances of performing cataract surgery in various ocular surface disorders and emphasize the need to have a comprehensive stepwise approach in such cases. In addition, they report the retrospective analysis of cataract surgery outcomes in 73 eyes of 57 patients with Stevens–Johnson syndrome.

*Miltos Balidis* *Miguel Rechichi* *Rajesh Fogla* *Zisis Gatzioufas*

